# A rare case of left transverse testicular ectopia in an adult

**DOI:** 10.1097/MS9.0000000000002747

**Published:** 2024-11-13

**Authors:** Suraj Keshari, Archana Pandey, Abiral Subedi, Rohit B. Shrestha, Priyanka Panta, Manisha Aryal, Ishwor Paudel, Abhishek Pandey, Prasamsa Pande

**Affiliations:** a Kathmandu University School of Medical Sciences, Dhulikhel Hospital, Dhulikhel; b Bharatpur Hospital, Bharatpur; c General Surgery, Bir Hospital, Kathmandu; d Dhulikhel Hospital, Dhulikhel, Kavre; e Kathmandu Medical College, Sinamangal, Kathmandu, Nepal

**Keywords:** case report, cryptorchidism, ectopic testis, transverse testicular ectopia, undescended testis

## Abstract

**Introduction and importance:**

Transverse testicular ectopia (TTE) is a rare congenital anomaly in which both testes descend into the same inguinal canal and are located in the same hemiscrotum. Diagnosing TTE can be challenging due to its rarity and unusual presentation.

**Case presentation:**

The authors present a case of a male in his 50s who was diagnosed with left transverse testicular ectopia after presenting with an empty right hemiscrotum and two testes-like masses in the left hemiscrotum.

**Clinical discussion:**

TTE usually presents with a history of an inguinal hernia and a contralateral undescended testis. However, in our case, the patient had both testes in the left hemiscrotum without an inguinal hernia, making this case even rarer.

**Conclusion:**

Patients presenting with an empty hemiscrotum on one side and two testes-like masses on the other should be suspected of having TTE. In such cases, it is important to use imaging like USG or MRI, to identify and locate the ectopic site and assess the testicular morphology.

## Introduction

Highlights

- Transverse testicular ectopia (TTE) is a rare congenital anomaly in which both testes descend through the same inguinal canal and are located in the same hemiscrotum.
- A typical clinical history of TTE includes an inguinal hernia and contralateral undescended testis.
- In this case, however, the patient had both testes in the left hemiscrotum without an inguinal hernia.

Occasionally, the testes fail to descend completely or may diverge from the typical course. The former disorder is known as undescended testis and affects ~4.5% of neonates, with preterm babies having a higher incidence, whereas the latter is known as testicular ectopia (TE)^1^. TE is a rare disorder in which the testes lie outside of the typical descent route (retroperitoneum to scrotum). TE has numerous variations, including perineal ectopic testis (PET), femoral and penile ectopic testis, transverse TE (TTE), and preperitoneal and anterior abdominal wall ectopic testis^2^. Transverse testicular ectopia is a rare congenital condition in which both testes descend through the same inguinal canal and are found in the same hemiscrotum^3^. Von Lenhossek reported it for the first time in 1886^4^. Transverse testicular ectopia (TTE) is estimated to have an incidence of 1 in every 4 million children^5^. Although the majority of instances are identified in children, there have also been reports of adult cases^3^. Similar to other dysgenetic testes, there is a considerable likelihood that they may proceed to malignancy^6^.

Here, we present a case report of a man in his 50s who presented to our tertiary care center with transverse testicular ectopia (TTE). We also share our experience with this condition and discuss the magnetic resonance imaging (MRI) findings in detail. This case is being reported for its rarity and to highlight the role of MRI in accurately diagnosing such uncommon conditions. This case has been reported in line with the Surgical CAse REport (SCARE) 2023 criteria^7^.

## Case presentation

A 50-year-old male was referred to our tertiary care hospital from a community hospital following incidental findings during evaluation for abdominal pain persisting for 5 days. A health professional examined him at the community hospital and found his right scrotal sac empty. However, palpation of his left scrotal sac revealed a palpable testis along with an additional soft, round mass. The patient’s abdominal pain led to the diagnosis of acid peptic disease; however, due to the suspicion of malignancy arising from the presence of this additional mass in the left hemiscrotum, he was referred for further evaluation. The patient then presented to our tertiary care hospital’s surgery outpatient department. Notably, the patient had been aware of the absence of testis in his right hemiscrotum since childhood but was not aware of the additional mass in his left hemiscrotum. He denies any history of trauma or pain in this scrotal area. He is the father of two children. Both medical and surgical histories are unremarkable. He reports no prior use of medications and has no known allergies. No similar conditions have been reported in the family. He had a history of occasional alcohol consumption but denied any history of smoking.

On examination, we found an uncircumcised penis with a urethral opening at a normal location, an empty right hemiscrotum and two testes-like masses in the left hemiscrotum. These masses were soft, non-tender, and were not fixed. The overlying skin of the scrotum was normal with no redness or swelling. There was no inguinal hernia. Systemic examination detected no abnormalities.

**Figure 1:**
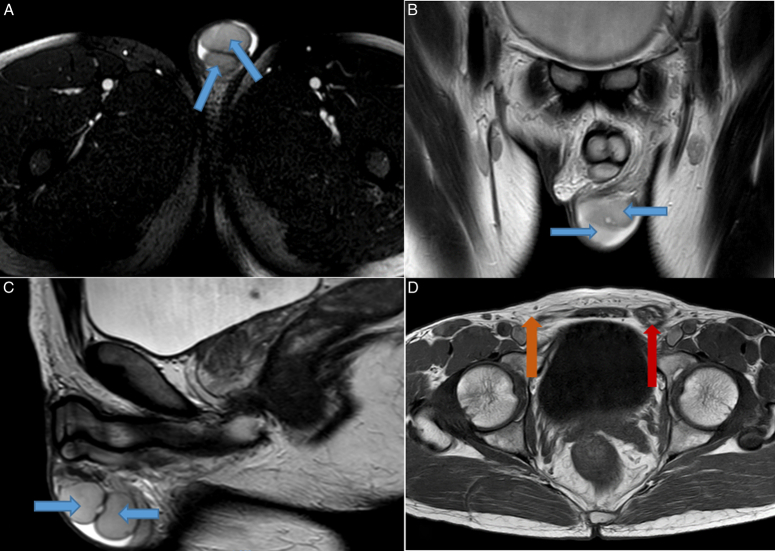
MRI images of scrotum and pelvis. (A) Axial T2W(SPIR) image shows two testes in the left hemiscrotum (blue arrows) and an empty right scrotal sac. (B, C) Coronal T2W and sagittal T2W images, respectively, show two testes in the left hemiscrotum (blue arrows). (D) Axial T2W image reveals fused left-sided vas deferens (red arrow), which divide to approach both testes and the absence of right-sided vas deferens (orange arrow).

Ultrasonography (USG) of the abdomen, pelvis, and scrotum was performed, which revealed an empty right hemiscrotum and two testes showing adequate vascular flow on the color Doppler within the left hemiscrotum. Bilateral inguinal and femoral hernial orifices were intact. Additionally, other abdominal organs appeared grossly intact. Following the USG findings, magnetic resonance imaging (MRI) of the pelvis was planned to confirm the diagnosis of TTE and to better understand the spatial relationships of the testes, vas deferens, and associated structures. MRI was also considered in this case due to the unusual presentation of this patient in his 50s, when the possibility of malignancy needed to be ruled out. MRI of the pelvic region (T2/SPIR sequences in axial, sagittal, and coronal planes) revealed two testes in the left hemiscrotum and an empty right hemiscrotum, and fused left-sided vas deferens, which divides within the left inguinal canal along its mid-aspect to approach both testes and the absence of right-sided vas deferens (Fig. 1).

A transseptal orchiopexy was offered to put back the ectopic testis in the right hemiscrotum, considering the future risk of malignancy. However, the patient refused. We plan to monitor the patient on a regular basis, with follow-ups every 6 months. Each follow-up will include a physical examination and ultrasonography of the scrotum.

## Discussion

TTE is also denoted as crossed testicular ectopia. Transverse testicular ectopia (TTE) is a rare case of testicular migration in which a testis descends into the opposite hemiscrotum. Approximately 260 cases of transverse testicular ectopia have been reported in the literature^8^. However, the mean age at presentation is 4–5 years. We have reported an adult diagnosed with TTE at the age of 50, which is quite uncommon for this condition. Another case report of a man diagnosed with TTE in adulthood stated that low socioeconomic status, lack of education, and lack of awareness may have led to delayed hospital presentation^9^. The ectopic testis might be in the abdomen, at the level of the internal inguinal ring, or in the same hemiscrotum^10^. In TTE, the ectopic testis lies in the same hemiscrotum. A typical clinical history of TTE includes an inguinal hernia and contralateral undescended testis^9^. However, these findings were not present in our case, which made it even rarer.

Many theories have been proposed to explain the development of TTE. According to Gupta and Das, adhesion and fusion of the developing Wolffian ducts took place early, and the descent of one testis caused the second one to follow^11^. Paltii *et al*. showed that obstruction of the inguinal ring or abnormalities in the implantation of the testicular gubernaculum may prevent the testicles from descending to the ipsilateral side^3^. Transverse testicular ectopia (TTE) can arise as a result of aberrant testicular adhesion to neighboring structures, as demonstrated by Josso *et al*.^11^ There is no one explanation that can explain TTE, despite the fact that many theories have been proposed.

TTE may occur alone or in conjunction with inguinal hernia, hypospadias, scrotal abnormalities, true hermaphroditism, and PMDS. However, in this case, TTE occurred alone and was not associated with any of these abnormalities. Based on the presence of different extra anomalies, transverse testicular ectopia is divided into three categories^12,13^.

1. Seen only with inguinal hernia (40–50%).
2. Reported with persistent or rudimentary Mullerian duct structures (30%).
3. Found to have other anomalies (20%) other than Mullerian remnants, such as inguinal hernia, hypospadias, pseudohermaphroditism, fused vas deferens, seminal vesicle cysts, and testicular microlithiasis.
  (4) Since this case involves fused vas deferens without an inguinal hernia, it belongs to type 3 transverse testicular ectopia.

When one or both testicles are palpable on the same side and the scrotum is empty on the other, a clinical diagnosis may be made in certain situations. Magnetic resonance imaging (MRI) or ultrasonography (USG) can be used for preoperative diagnosis. On ultrasonography, TTE has two testes with similar echotexture and vascular flow patterns to a normal testis on one side and an empty hemiscrotum on the other^14^. We found similar USG findings in this case, showing two normal-looking testes with adequate vascular flow in the left hemiscrotum and an empty right hemiscrotum.

Magnetic resonance imaging (MRI) and magnetic resonance venography (MRV) have 82.4% and 100% sensitivity in detecting TTE, respectively^6^. According to a study by Lam *et al*.^6^, if USG results are inconclusive, MRI should be done next, and if MRI results are also inconclusive, MRV should be done. MRI was used as a supplementary tool due to the unusual presentation of this patient in his 50s, when the possibility of malignancy needed to be ruled out. In TTE, both testes show a normal appearance on MRI. They show intermediate signal intensity on T1-weighted imaging and high intensity on T2-weighted imaging. Other structures of the anomaly, such as associated hernia or fusion of the vas deferens, can also be visualized in MRI^14^. Similar findings were observed on the MRI scan in this case. The combination of clinical findings, along with USG and MRI findings, led to the diagnosis of TTE in this case.

Transseptal orchiopexy and transperitoneal orchiopexy are the surgical procedures used for the management of TTE^15^. The transseptal orchiopexy technique involves moving the ectopic testis to the other side of the scrotal septum through a window that is formed in the septum. The transperitoneal orchiopexy technique involves moving the ectopic testis into the extraperitoneal space by crossing the root of the penis and fixing it into the opposite side of the hemiscrotum. However, the testicular vessels and vas deferens need to be long enough for this procedure^15^. Additionally, laparoscopy is a procedure that is helpful for treating and diagnosing TTE and related abnormalities^16^.

## Strengths and limitations

This case report explains the clinical presentation and imaging findings in a patient with transverse testicular ectopia. However, given the unusual presentation in adulthood, the presence of fused vas deferens, and the absence of an inguinal hernia, the findings may not be applicable to other patients with similar conditions, particularly those presenting in childhood, or with different associated anatomical variations. Such variations in clinical presentation, including different anomalies or associated conditions, influence both diagnosis and treatment approaches. Therefore, the results may not be generalizable to all cases. Furthermore, this case report does not contain long-term follow-up.

## Conclusion

Patients who present with an empty hemiscrotum on one side and two testes-like masses on the other should be suspected of having TTE. In suspected cases of TTE, it is important to use preoperative imaging (USG and MRI) to identify and locate the ectopic site, assess the testicular morphology, and detect associated anomalies. Early diagnosis of TTE improves the chances of fertility, conserving the ectopic testis, screening for malignancy, and planning surgical intervention accordingly. The usual approach for diagnosing TTE begins with a USG examination; if the results are not conclusive, an MRI can be performed; if the results of the MRI are likewise not conclusive, an MRV can be performed.

## Ethical approval

Not applicable.

## Consent

Written informed consent was obtained from the patient for publication of this case report and accompanying images. A copy of the written consent is available for review by the Editor-in-Chief of this journal on request.

## Source of funding

Not applicable.

## Author contribution

S.K.: concept, obtaining consent, performing radiodiagnosis, manuscript preparation, edit, and review; A.P., P.P., M.A., I.P., A.P., and P.P.: manuscript preparation, edit, and review; A.S.: manuscript preparation, data collection, manuscript edit, and review; R.B.S.: data collection, manuscript preparation, edit, and review.

## Conflicts of interest disclosure

The authors declare no conflict of interest.

## Research registration unique identifying number (UIN)

1. Name of the registry: not applicable.
2. Unique identifying number or registration ID: not applicable.
3. Hyperlink to your specific registration (must be publicly accessible and will be checked): not applicable.

## Guarantor

Suraj Keshari.

## Data availability statement

Not applicable.

## Provenance and peer review

Not commissioned, externally peer-reviewed.
